# Neuromuscular strain as a contributor to cognitive and other symptoms in chronic fatigue syndrome: hypothesis and conceptual model

**DOI:** 10.3389/fphys.2013.00115

**Published:** 2013-05-16

**Authors:** Peter C. Rowe, Kevin R. Fontaine, Richard L. Violand

**Affiliations:** ^1^Division of General Pediatrics and Adolescent Medicine, Department of Pediatrics, Johns Hopkins University School of MedicineBaltimore, MD, USA; ^2^Department of Health Behavior, University of Alabama School of Public HealthBirmingham, AL, USA; ^3^Violand and McNerney, PAEllicott City, MD, USA

**Keywords:** adverse neural tension, neurodynamics, chronic fatigue syndrome, manual therapy, central sensitivity, orthostatic intolerance

## Abstract

Individuals with chronic fatigue syndrome (CFS) have heightened sensitivity and increased symptoms following various physiologic challenges, such as orthostatic stress, physical exercise, and cognitive challenges. Similar heightened sensitivity to the same stressors in fibromyalgia (FM) has led investigators to propose that these findings reflect a state of central sensitivity. A large body of evidence supports the concept of central sensitivity in FM. A more modest literature provides partial support for this model in CFS, particularly with regard to pain. Nonetheless, fatigue and cognitive dysfunction have not been explained by the central sensitivity data thus far. Peripheral factors have attracted attention recently as contributors to central sensitivity. Work by Brieg, Sunderland, and others has emphasized the ability of the nervous system to undergo accommodative changes in length in response to the range of limb and trunk movements carried out during daily activity. If that ability to elongate is impaired—due to movement restrictions in tissues adjacent to nerves, or due to swelling or adhesions within the nerve itself—the result is an increase in mechanical tension within the nerve. This adverse neural tension, also termed neurodynamic dysfunction, is thought to contribute to pain and other symptoms through a variety of mechanisms. These include mechanical sensitization and altered nociceptive signaling, altered proprioception, adverse patterns of muscle recruitment and force of muscle contraction, reduced intra-neural blood flow, and release of inflammatory neuropeptides. Because it is not possible to differentiate completely between adverse neural tension and strain in muscles, fascia, and other soft tissues, we use the more general term “neuromuscular strain.” In our clinical work, we have found that neuromuscular restrictions are common in CFS, and that many symptoms of CFS can be reproduced by selectively adding neuromuscular strain during the examination. In this paper we submit that neuromuscular strain is a previously unappreciated peripheral source of sensitizing input to the nervous system, and that it contributes to the pathogenesis of CFS symptoms, including cognitive dysfunction.

## Introduction

Chronic fatigue syndrome (CFS) affects an estimated 1 million individuals in the United States. The quality of life in adults with CFS can be comparable to that for individuals with congestive heart failure or multiple sclerosis (Komaroff et al., 1996). In adult studies, spontaneous recovery is uncommon if CFS has been present for over 3 years (Jason et al., 1999; Cairns and Hotopf, 2005). Among adolescents, CFS is it is one of the most common causes of prolonged school absence (Smith et al., 2003; Crawley et al., 2011). Cognitive-behavioral therapy and graded exercise are the treatments best supported by the evidence, but treatment effect sizes are modest and these treatments offer suboptimal relief for those with more profound illness (Price et al., 2008). Thus, CFS has substantial personal, medical, and societal costs (Jason et al., 2008), underscoring the need for better understanding of pathophysiology and more effective treatments.

## Central sensitivity as a conceptual model for CFS

Individuals with CFS have heightened sensitivity and increased symptoms following various physiologic challenges, such as orthostatic stress, physical exercise, and cognitive challenges. Similar heightened sensitivity to the same stressors in FM has led investigators to propose that these findings reflect a state of central sensitivity. As defined by Yunus, central sensitivity is “clinically and physiologically characterized by hyperalgesia (excessive sensitivity to a normally painful stimulus, e.g., pressure), allodynia (painful sensation to a normally non-painful stimulus, e.g., touch and massage), expansion of the receptive field (pain beyond the area of peripheral nerve supply), prolonged electrophysiological discharge, and an after-stimulus unpleasant quality of pain (e.g., burning, throbbing, numbness)” (Yunus, 2008). This has obvious relevance for the pain symptoms in CFS and for FM. Other related models propose that CFS represents a state of altered homeostasis characterized by sustained arousal akin to a permanent stress response (Wyller et al., 2009).

## Gaps in the central sensitivity model for CFS and FM

A large body of evidence supports the concept of central sensitivity in FM (Yunus, 2005; Jason et al., 2008; Albin and Clauw, 2009) and despite the estimated 35–70% clinical overlap between the disorders in adults (White et al., 2000; Brown and Jason, 2007), a more modest literature provides partial support for this model in CFS, particularly with regard to pain (Vecchiet et al., 1996; Whiteside et al., 2004; Meeus et al., 2009). However, the fatigue and cognitive dysfunction found in CFS and FM “cannot be satisfactorily explained” (Yunus, 2008) by the central sensitivity data thus far (Geisser et al., 2007). These symptoms might be mediated by amplified central sensitivity, but peripheral factors, which have been described in FM and irritable bowel syndrome (IBS), may also play a role (e.g., Price et al., 2009; Staud et al., 2009). Staud has shown that local anesthetic injection into trapezius muscle tender points results in lower levels of thermal hyperalgesia in the forearm, consistent with peripheral nociceptive input as a contributor to central sensitization (Staud et al., 2009). Others have confirmed and extended these findings in subjects with FM (Affaitati et al., 2011; Alonso-Blanco et al., 2011), but these studies have focused on pain. No data have addressed whether non-pain symptoms such as fatigue or cognitive dysfunction also have peripheral contributors.

## How might neuromuscular strain be a peripheral influence on central sensitivity?

A series of observations over the last several decades—by Brieg, Sunderland, and others (Lindquist et al., 1973; Brieg, 1978; Sunderland, 1978; Butler, 1991, 2000; Kornberg and McCarthy, 1992; Shacklock, 1995; Slater and Wright, 1995; Elvey, 1997; Rempel et al., 1999; Orlin et al., 2005; Topp and Boyd, 2006)—has focused attention on the ability of the nervous system to undergo accommodative changes in length in response to the range of limb and trunk movements carried out during daily activity. The interaction of nerve mechanics and function has been termed neurodynamics. As an example of the principles of neurodynamics, the median nerve elongates approximately 20% as the upper extremity moves from a position of full wrist and elbow flexion to one of full wrist and elbow extension (Butler, 1991). If that ability to elongate is impaired—due to movement restrictions in tissues adjacent to the median nerve and its branches, or due to swelling or adhesions within the median nerve itself—the result is an increase in mechanical tension within the nerve. This adverse neural tension, also termed neurodynamic dysfunction, is thought to contribute to pain and other symptoms through mechanical sensitization and altered nociceptive signaling, altered proprioception, adverse patterns of muscle recruitment and force of muscle contraction, reduced intra-neural blood flow, and release of inflammatory neuropeptides (Lindquist et al., 1973; Kornberg and McCarthy, 1992; Shacklock, 1995; Slater and Wright, 1995; Balster and Jull, 1997; Van der Heide et al., 2001; Kobayashi et al., 2003; Orlin et al., 2005). It is now well-established that manual stretch of nerves is capable of evoking increased sweating and alterations of blood flow in peripheral tissues, providing evidence of electrophysiologic activity in sympathetic nerve fibers (Lindquist et al., 1973; Kornberg and McCarthy, 1992; Slater and Wright, 1995; Orlin et al., 2005). Conversely, treatment of areas of adverse neural tension (for example in carpal tunnel syndrome, cervico-brachial pain, and osteoarthritis) leads to improved functional outcomes (Rozmaryn et al., 1998; Deyle et al., 2000; Tal-Akabi and Rushton, 2000; Akalin et al., 2002; Allison et al., 2002).

Certain “neural provocation” maneuvers can assess for adverse tension and other dysfunctions within the neuromuscular system, including altered range of motion, altered resting muscle tone, and hyperalgesia along the course of the involved nerve tissue (Elvey, 1997; Butler, 1991, 2000). The most notable examples of these provocation maneuvers are ankle dorsiflexion, the passive straight leg raise test, the upper limb tension (or neurodynamic) tests, and the seated slump test (Butler, 1991, 2000). Test-retest reliability is good for straight leg raise, slump testing, and upper limb neurodynamic testing. (Coppieters et al., 2001; Herrington et al., 2008) Because it is not possible to differentiate completely between adverse neural tension and strain in muscles, fascia, and other soft tissues, we will use the more general term “neuromuscular strain” in this paper. The concepts and clinical maneuvers described above, while somewhat foreign to physicians and usually not part of current medical school training, are nonetheless widely accepted in the physical therapy literature. (Topp and Boyd, 2006).

## Conceptual model: neuromuscular strain as a peripheral propagator of central sensitization (Figure 1)

**Figure 1:**
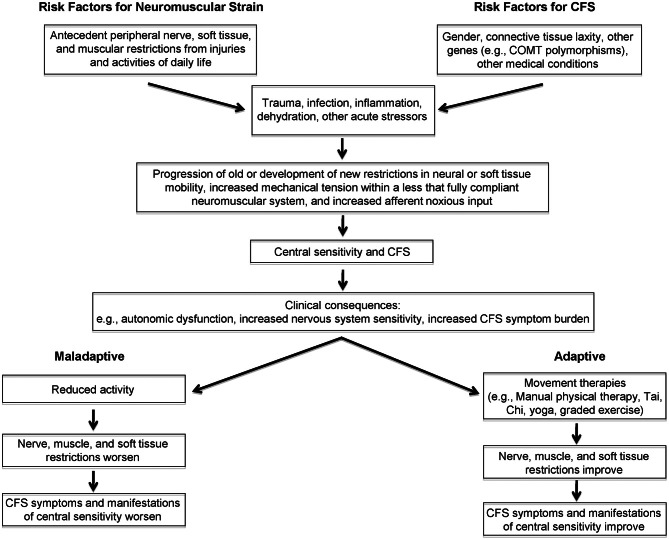
**Conceptual model linking peripheral, afferent input to central sensitivity and symptom expression in chronic fatigue syndrome**.

We propose that peripheral neuromuscular factors contribute to the heightened perception of physiologic signals in CFS. As shown on the left in Figure 1, neuromuscular strains and movement restrictions can develop as a result injuries and activities of daily life (for example, due to soft tissue and peri-neural adhesions around scars, contusions and fractures that reduce range of motion, anatomic abnormalities like scoliosis and kyphosis, overuse injuries, and others). Their prevalence and severity is likely modulated by the individual's connective tissue phenotype or general flexibility, the level of habitual exercise or the attention to proper rehabilitation of injuries, and whether maladaptive activities such as overuse are corrected. A number of genetic factors predispose individuals to symptoms of CFS, including (though not limited to) polymorphisms in the genes controlling catechol-O-methyltransferase activity [as shown recently in CFS by Sommerfeldt and colleagues (2011)], and connective tissue laxity (Rowe et al., 1999; Barron et al., 2002). Gender is an important predisposing factor, given that many more women than men develop CFS, although the mechanism for the increased risk is not known.

In response to a new stressor—examples of which include trauma, surgery, infection, dehydration, and others—a variety of physiologic changes occur, prominent among them cytokine alterations in response to infection and inflammation. While these stressors could be sufficient to trigger CFS symptoms and central sensitivity, other biomechanical and behavioral factors—such as whether the individual rests or remains relatively active—modulate the response to a new stressor. For example, as has been demonstrated in experiments involving prolonged inactivity, reductions in plasma volume associated with long periods of bed rest (Fortney et al., 1996) would be expected to affect orthostatic tolerance (Bou-Holaigah et al., 1995; Rowe et al., 1995, 2001; Cordero et al., 1996; Freeman and Komaroff, 1997; Stewart et al., 1998; Schondorf et al., 1999; Stewart, 2000; Streeten et al., 2000; Newton et al., 2007; Wyller et al., 2007a,b; Jones et al., 2011). In those at risk for central sensitivity syndromes, these changes in response to a new stressor could give rise to progression of old (or the development of new) muscular, neural, and other soft tissue restrictions. These added movement restrictions would place further mechanical tension on an already less than fully compliant neuromuscular system. We hypothesize that this would result in increased noxious afferent input from the irritable peripheral tissues, thereby contributing to further central sensitization. Central sensitization, in turn, could aggravate peripheral factors including resting muscle tone, vascular and autonomic tone, and neural irritability. The peripheral factors, central sensitization, and orthostatic intolerance would then contribute to further expression of CFS symptoms. If the neuromuscular strains were not treated, and if the individual adapted to the increased symptom burden with decreased activity, then neural, soft tissue and muscular restrictions would be expected to worsen, leading to greater impairment and greater central sensitization. Conversely, this dynamic interplay between symptoms and further peripheral and central sensitization lends itself to potential interventions directed at (a) improving peripheral movement restrictions, via interventions such as manual physical therapy, exercise-based approaches, or therapies such as yoga or Tai Chi (Wang et al., 2010). Although not included in the proposed model, other ways of addressing central sensitivity are not excluded from this interplay. For example, improving central sensitivity—through addressing autonomic symptoms with treatment of orthostatic intolerance, or through improving central responses to stimuli via cognitive behavioral therapy, SSRI/SNRI medications, and anti-convulsant medications such as pregabalin—might allow improved exercise and might improve the response to movement therapies.

## Preliminary studies

In our clinical work, we have found that neuromuscular restrictions are common in CFS. A 2 year cohort study of 55 adolescent and young adult subjects with CFS is underway to more formally document the prevalence and impact of these restrictions compared to healthy controls, and to ascertain whether improvement in overall CFS symptoms is accompanied by improvement in the neuromuscular restrictions.

**Figure 2:**
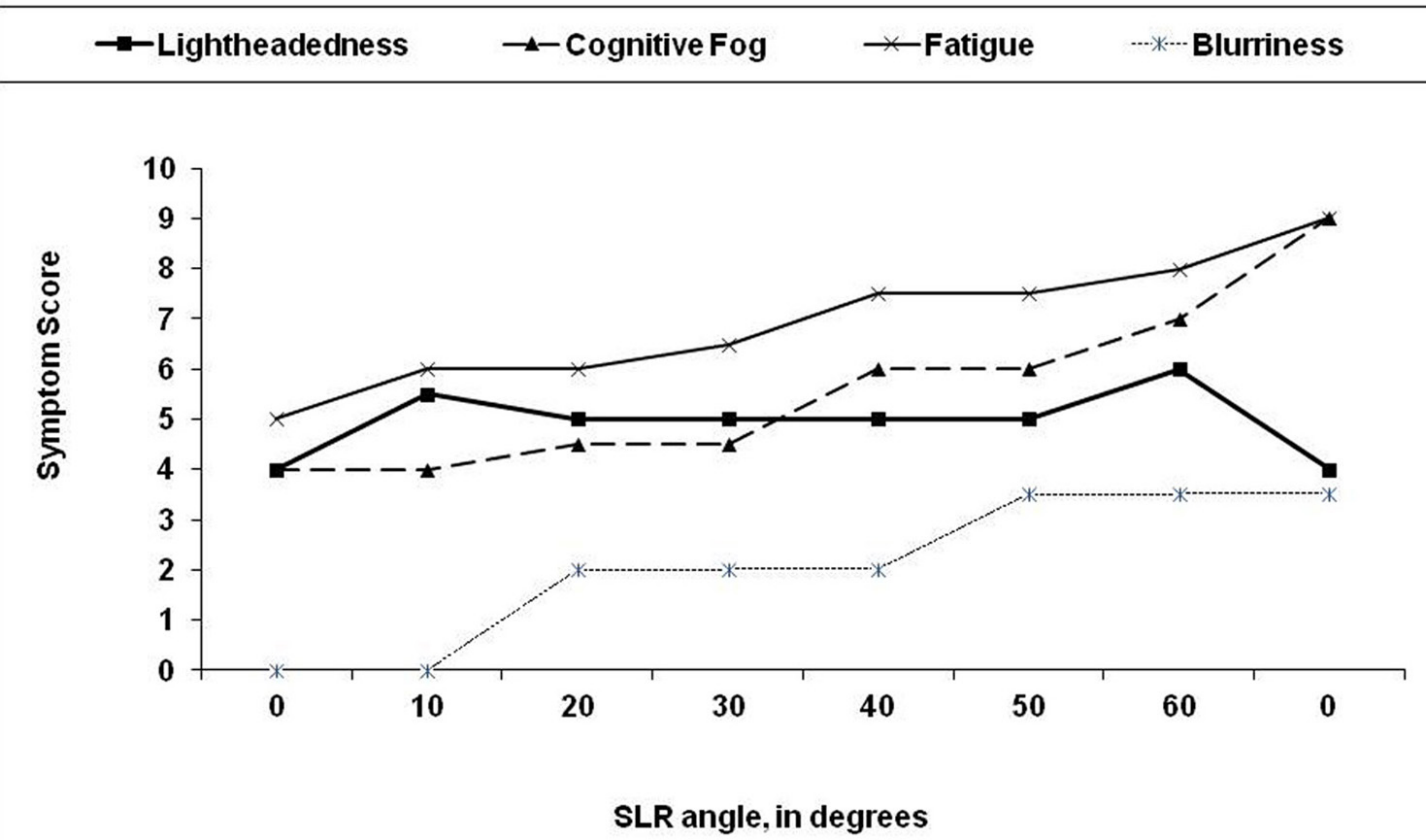
**Effect of 12 min of progressive passive straight leg raise (SLR) on symptom severity in a 19 year old man with chronic fatigue syndrome.** The leg was passively raised by 10 degree every 2 min. At the end of each 2 min period, the subject was asked to rate his symptoms on a 0–10 scale. After 12 min, the leg was returned to the horizontal plane.

We have also noted that many symptoms of CFS can be reproduced by selectively adding neuromuscular strain during the examination (Rowe et al., 2013a,b). As an illustration of the latter, two young adult males with CFS were placed supine and a sustained passive straight leg raise (SLR) was performed. A therapist held one leg elevated in SLR at 10° of hip flexion for 2 min, then raised it to 20° for 2 min, adding 10° incremental increases in SLR every 2 min until the 12 min point, at which time the leg was returned to the horizontal resting position. The responses to SLR were similar for both individuals, and so in Figure 2 we illustrate the symptom responses to progressive SLR in one subject. During the period of study, blood pressure, heart rate, skin temperature, and pulse oximetry remained stable, but both young men became progressively more symptomatic. After 12 min, they had difficulty answering basic questions. Symptoms were scored on a 0–10 scale; cognitive fogginess increased from 4/10 at baseline to 9/10 at the completion of the test. Despite the elevation of the leg, which might have been expected to improve venous return to the heart and thereby improve blood flow to the brain, lightheadedness increased, as did visual blurring. Both individuals remained more fatigued than usual for 12–24 h. Thus, supine neuromuscular strain provoked increased fatigue and cognitive disturbance, the two symptoms not adequately explained by the central sensitivity hypothesis thus far.

Such a dramatic change is not always present, and some subjects with CFS have no neuromuscular strains on examination. Nonetheless, the example above illustrates the ability of neuromuscular strain to provoke symptoms, and warrants further exploration to determine the prevalence of the problem, its overall contribution to symptoms, and the mechanisms by which neuromuscular strains increase symptoms. We have observed that open treatment of these movement restrictions using manual therapy is associated with clinical improvement (Rowe et al., 2013a,b).

The hypothesis can be tested by evaluating the whether the response to a given neuromuscular strain differs between CFS subjects and controls with regard to immediate and delayed (24-h) symptoms, and with regard to measures of central sensitivity, such as changes in heart rate variability, or changes in pain sensitivity as measured by pressure-pain thresholds. Further work will be needed to determine which neuromuscular strains are most prevalent, and whether specific areas of neurodynamic dysfunction are more associated with one group of symptoms or another. Moreover, it will be important to learn which neuromuscular strain paradigms are most likely to elicit symptoms in those with CFS, or whether individual variation in range of motion will require individualized strain maneuvers. A potential scientific challenge concerns the inability to determine whether any changes in symptoms are due to neural strain or to muscle stretch, but establishing whether and how often peripheral neuromuscular strain in general is capable of increasing symptoms will be required first before attempting to isolate whether neural or muscular factors predominate.

### Conflict of interest statement

The authors declare that the research was conducted in the absence of any commercial or financial relationships that could be construed as a potential conflict of interest.
